# Mesenchymal Stem Cell Derived Extracellular Vesicles in Aging

**DOI:** 10.3389/fcell.2020.00107

**Published:** 2020-02-21

**Authors:** Jérémy Boulestreau, Marie Maumus, Pauline Rozier, Christian Jorgensen, Danièle Noël

**Affiliations:** ^1^Institute of Regenerative Medicine and Biotherapies (IRMB), University of Montpellier, INSERM, Montpellier, France; ^2^Clinical Immunology and Osteoarticular Diseases Therapeutic Unit, Department of Rheumatology, CHU, Montpellier, France

**Keywords:** mesenchymal stem cells, extracellular vesicles, regenerative medicine, aging, senescence, clinical translation

## Abstract

Aging is associated with high prevalence of chronic degenerative diseases that take a large part of the increasing burden of morbidities in a growing demographic of elderly people. Aging is a complex process that involves cell autonomous and cell non-autonomous mechanisms where senescence plays an important role. Senescence is characterized by the loss of proliferative potential, resistance to cell death by apoptosis and expression of a senescence-associated secretory phenotype (SASP). SASP includes pro-inflammatory cytokines and chemokines, tissue-damaging proteases, growth factors; all contributing to tissue microenvironment alteration and loss of tissue homeostasis. Emerging evidence suggests that the changes in the number and composition of extracellular vesicles (EVs) released by senescent cells contribute to the adverse effects of senescence in aging. In addition, age-related alterations in mesenchymal stem/stromal cells (MSCs) have been associated to dysregulated functions. The loss of functional stem cells necessary to maintain tissue homeostasis likely directly contributes to aging. In this review, we will focus on the characteristics and role of EVs isolated from senescent MSCs, the potential effect of MSC-derived EVs in aging and discuss their therapeutic potential to improve age-related diseases.

## Introduction

Aging of the global population represents a growing burden on our healthcare system with a significant increase in the incidence of co-morbidities, including neurodegeneration, diabetes, cardiovascular diseases, cancer, osteoporosis, and osteoarthritis (OA), among others (Suzman et al., 2015). It is expected that in the coming years, people aged 65 years and over will outnumber children younger than 5 years. The rise of degenerative chronic diseases in the elderly not only influences negatively their quality of life but impacts financially our social security systems. There is therefore an urgent need to better understand the mechanisms driving aging and how we can positively impact on age-related disorders to develop novel therapeutic strategies.

## Aging and Senescence

Aging is a complex process resulting from the accumulation of unpredictable molecular and cellular alterations in a time-dependent manner. Aging is characterized by nine hallmarks: genomic instability, telomere attrition, epigenetic alteration, loss of proteostasis, metabolic dysfunction, mitochondrial dysfunction, cellular senescence, stem cell exhaustion, and altered intercellular communications (for review see Lopez-Otin et al., 2013; Lidzbarsky et al., 2018). Subsequently, aging is a cell-autonomous accumulation of damages to organelles and macromolecules in cells and organs. Cell non-autonomous mechanisms also play a role in modulating the degenerative changes occurring spontaneously. As examples, the pro-geronic C-C motif chemokine ligand 11 (CCL11) detected in serum from old mice can drive aging in young mice (Villeda et al., 2011). By contrast, the anti-geronic growth and differentiation factor (GDF11) identified in serum from young mice can induce rejuvenation after transfer in old mice using heterochronic parabiosis (Katsimpardi et al., 2014). However, the anti-aging role of GDF11 has been questioned since a potent inhibitory effect on skeletal muscle regeneration has been described thereafter (Brun and Rudnicki, 2015; Egerman et al., 2015). Although circulating factors may be important actors in the maintenance and propagation of aging, the identification of such factors is still missing. By contrast, the possibility that circulating EVs may instead mediate the beneficial function of a young milieu has been reported in a couple of studies and recently discussed (Prattichizzo et al., 2019).

**FIGURE 1:**
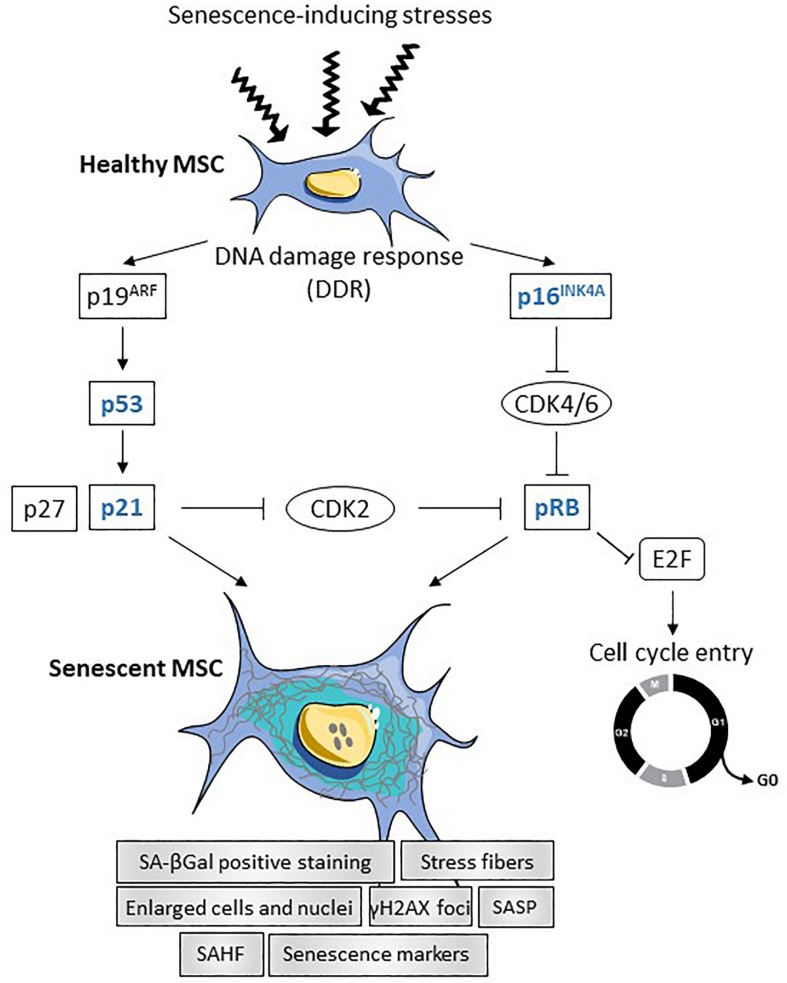
Summary of transcriptional regulation of senescence in MSCs. In response to different stresses (oncogene, telomere attrition, oxydative stress, inflammation or mitochondrial dysfunction), DNA damage response (DDR) is induced in healthy MSCs leading to the activation of the two main signaling pathways p19^*ARF*^ and p16^*INK*4*A*^. Activation of p19^*ARF*^ results in p53 and p21 activation, which inhibits CDK2 and induces senescence. Activation of the p16^*INK*4*A*^-pRB pathway leads to cell cycle arrest and triggers senescence. Senescent MSCs are characterized by enlarged cells and nuclei, increased number of stress fibers, increased number of γH2AX foci and senescence-associated heterochromatin foci (SAHF), positive staining for SA-βGal and increased senescence markers and senescence-associated secretory phenotype (SASP).

Cellular senescence is characterized by permanent cell cycle arrest with resistance to cell death through necrosis, apoptosis or autophagy. It can be seen as a cell defense mechanism preventing unwanted proliferation of damaged cells to proceed toward oncogenic transformation. Senescence is likely not a single cell state and recent evidence highlights that distinct stimuli can induce different modes of senescence (for review, see Lunyak et al., 2017). DNA damage is a key inductive factor of senescence induced by physical (irradiation) or chemical [reactive oxygen species (ROS), mutagens] stress stimuli (*stress-induced senescence*). The imbalance between the production of ROS and anti-oxidants defined as oxidative stress contributes not only to DNA damage but also to protein damage and mitochondrial dysfunction leading to loss of homeostasis and senescence (Li et al., 2017). Other well characterized causes of senescence are telomere shortening (*replicative senescence*) and oncogene activation (*oncogene-induced senescence, OIS*), which also lead to DNA damage and persistent DNA damage response (DDR). Recently, the importance of senescence induced by transforming growth factor-β (TGF-β)/SMAD and phosphoinositide 3-kinase (PI3K)/Forkhead box O (FOXO) pathways has been highlighted as part of the normal developmental process (*developmental senescence*) (Munoz-Espin et al., 2013). Whatever the inductive signal, senescence is primarily controlled by the p53/p21CIP1 (p21) and p16INK4A/pRB signaling pathways (Larsson, 2011). Stimuli activating the DDR trigger the transcriptional activation of p53 inducing the cyclin-dependent kinase inhibitor (CDKI) p21, which inhibits pRB phosphorylation and prevents E2F activity (Figure 1). Expression of p16INK4A also leads to pRB activation and E2F inactivation. Depending on the stimulus, either or both pathways may be activated. Furthermore, senescence may be transient or chronic. Transient senescence is involved in a beneficial process for normal development and regeneration while chronic senescence is associated with harmful process leading to disease and aging.

Senescent cells are characterized by enlarged nuclei and flattened morphology with presence of stress fibers, decreased adherence on plastic and vacuolization resulting from the dysregulated accumulation of macromolecules. They stain positive for acidic senescence-associated β-galactosidase (SA-βGal) activity, which is one of the most used senescence markers. More recently, senescence-associated lysosomal α-L-fucosidase (SA-α-Fuc) was proposed as a more sensitive and robust biomarker for cell senescence as compared to SA-βGal (Li et al., 2017). The presence of senescence-associated heterochromatin foci (SAHF), which are stained by DAPI, and the expression of lysine 9-trimethylated histone H3 (H3K9Me3) are also hallmarks of DRR-associated senescence. The activity of the DDR can be highlighted by revealing the accumulation of γH2AX protein in the nucleus (Larsson, 2011). Markers of senescent cells include enhanced expression of cell cycle regulators (p16INK4a, p21, p27, p53, pRB) and of senescence-associated secretory phenotype (SASP) factors (Figure 1). The SASP comprises cytokines [interleukin-1β (IL-1β), IL-6], chemokines [monocyte chemoattractant protein-1 (MCP-1), IL-8], growth factors [vascular endothelial growth factor (VEGF), basic fibroblast growth factor (bFGF), hepatocyte growth factor (HGF), insulin growth factor-1 (IGF-1), TGFβ] and extracellular proteases [matrix metalloprotease-1 (MMP-1), -3, -13]. Of note, a large number of these factors are expressed at high basal levels in multipotent mesenchymal stromal or stem cells (MSCs).

## Mesenchymal Stem Cells and Extracellular Vesicles

Mesenchymal stem cells (MSCs) are isolated in large quantities from different tissue sources, especially bone marrow, adipose tissue or perinatal tissues and can be expanded *ex vivo*. They are defined by their capacity to adhere to plastic, a set of phenotypic markers (CD73^+^, CD90^+^, CD105^+^, CD11b^–^ or CD14^–^, CD19^–^ or CD79a^–^, CD34^–^, CD45^–^, HLA-DR^–^) and the ability to differentiate toward osteoblasts, adipocytes and chondrocytes (Dominici et al., 2006). In addition to their differentiation potential, MSCs are supportive cells exerting pleiotropic functions. They can exert namely anti-inflammatory, pro-proliferative, pro-angiogenic, anti-fibrotic, anti-apoptotic functions thanks to cell-cell interactions and secretion of a high number of soluble molecules. Among these, prostaglandin E2 (PGE2), TGFβ, IL-6, IL-1 receptor antagonist (IL-1RA), tumor necrosis factor (TNF)-inducible gene 6 protein (TSG6), nitric oxide (NO) produced by inducible NO synthase (iNOS) or kynurenine produced by indoleamine 2. 3-dioxygenase (IDO) are part of the anti-inflammatory secretome (Harrell et al., 2019). Other molecules such as HGF, FGF, VEGF are important components of the paracrine activity of MSCs. These molecules act upon release in the extracellular compartment but recent evidence indicates that they are primarily conveyed within extracellular vesicles (EVs) that play a key role in the cell-cell communication pathways.

Extracellular vesicles are divided into two main subtypes: exosomes and microvesicles (or microparticles) (van Niel et al., 2018). Exosomes are small particles (less than 120 nm) originating from the endosomal compartment and produced in multivesicular bodies (MVB) that thereafter fuse with the plasma membrane to release their exosomal content. Microvesicles are vesicles around 100–500 nm that form by the budding of the plasma membrane under stress-inductive conditions. A third type of EVs called apoptotic bodies are characterized by a larger size (500–5000 nm) and a release upon fragmentation of apoptotic cells. However, they are not in the focus of the present review. The heterogeneity in EV size makes difficult to separate exosomes from microvesicles using current procedures for EV isolation, essentially based on physico-chemical properties. Because large exosomes and small microvesicles share similar density and size, the available purification methods can only separate small size and large size EVs (sEVs and lEVs, respectively), independently of their biogenesis (Thery et al., 2018). EVs contain proteins, lipids and nucleic acids, including mRNA, DNA and non-coding RNAs such as microRNAs and long non-coding RNAs (lncRNAs). They are enriched in some specific proteins (tetraspannins, members of the endosomal sorting complexes required for transport (ESCRT), heat shock proteins) and lipids, especially cholesterol and sphingolipids. Nonetheless, the content of EVs mirrors the cell of origin and EVs also convey molecules that are specific for a particular cell type.

Extracellular vesicles are found in all biological fluids (blood, urine, breast milk, saliva, cerebrospinal fluid, synovial fluid,…) and are produced by all cell types. They are recognized as important components of the cell-to-cell communication pathways and exert a number of functions depending upon the parental cell and the environmental context. They are involved in tissue homeostasis maintenance and repair. They may also reflect the pathological state of the releasing cells, making them promising biomarkers of diverse pathologies (Svenningsen et al., 2019). EVs are therefore attractive as biomarkers for diagnosis or prognosis purposes and as therapeutic agents with documented activities related to the parental cell origin. EVs isolated from MSCs (MSC-EVs) are of particular interest for the scientific community because they reproduce the main functions of the parental cell, notably their immunosuppressive effect, and are safer since they do not possess nuclei and cannot replicate (Baldari et al., 2017). However, the function and the number of MSCs are altered with aging, which likely impact the content and effect of MSC-EVs (Li et al., 2017; Robbins, 2017). This dysfunction contributes to a large extend to the age-related degenerative changes in old individuals.

## Effect of Aging on MSCs

Although data on the functionality of MSCs isolated from aged subjects with respect to young individuals are still debated in the literature, some consensual evidence appears. With the increase of donor age, MSCs from bone marrow are reported to show a decrease in proliferative and clonogenic/self-renewal capacities, characterized by the number of colony-forming unit-fibroblasts (CFU-F) but no phenotypic change is correlated with age (for review, see Charif et al., 2017). Nevertheless, a low expression level of CD146 is associated with late passages and shortening of telomeres in MSCs (for review, see Fafian-Labora et al., 2019). In addition, a decrease in the expression of CD106 and Stro-1 is observed in late passage MSCs while CD295 (leptin receptor) is increased (for review, see Li et al., 2017). Functionally, the differentiation capacity of MSCs, notably the osteogenic and chondrogenic differentiation potential, decreases with increasing donor age as well as their capacity to polarize macrophages toward the anti-inflammatory M2 phenotype (Yin et al., 2017). Adipogenic differentiation of MSCs is reported to increase with age. Interestingly, autophagy is increased in MSCs entering replicative aging (for review, see Fafian-Labora et al., 2019). Autophagy may play a dual role: in some models, autophagy induction is required for senescence while in other contexts, the decrease of autophagy induces senescence (Falser et al., 1987; Capasso et al., 2015). Nonetheless, the current paradigm underlines a key role for autophagy in reversing partially the senescence process occurring during aging (for review, see Fafian-Labora et al., 2019). Finally, levels in ROS and resulting oxidative stress are increased in aging MSCs (Marycz et al., 2016). Oxidative stress may result from low grade chronic inflammation occurring in aging and many degenerative diseases (Tofino-Vian et al., 2017). By contrast, treatment of MSCs with melatonin protects them from oxidative stress and related senescence highlighting the correlation between oxidative stress and senescence (Yun et al., 2018).

Replicative senescence can be induced in MSCs upon passages within population doublings (PD) 20–50 in culture. MSCs stop proliferating but maintain their metabolic state for a prolonged time. Although telomere shortening is not detected, accumulation of DNA damages and activation of the DDR together with loss of epigenetic control on chromatin deterioration have been described (for review, see Lunyak et al., 2017). MSCs have also been shown to enter OIS in response to oncogene exposition or loss of tumor suppressor genes (Braig et al., 2005). They can enter stress-induced senescence when exposed to oxidative stress, doxorubicin, bleomycin or very low doses of pesticides or irradiation (for review, see Lunyak et al., 2017). Finally, although not shown yet in MSCs, ectopic expression of the four reprogramming transcription factors OCT4, SOX2, KLF4 and C-MYC (OSKM) can cause senescence, which suggests that it could be indispensable for organism development (Mosteiro et al., 2016). Indeed, aging and senescence impact MSC characteristics in several ways and affect also the release of bioactive factors and EVs. A detailed analysis of the MSC secretome in different models of induced senescence revealed different protein profiles sustaining different expected functions (for review, see Lunyak et al., 2017).

Interestingly, a couple of studies indicate that age-related alterations in MSCs can be reversed. One interesting study reports a significant increase of cell division cycle 42 (Cdc42) activity in aged MSCs that can be decreased by the selective inhibitor ML141 (Chaker et al., 2018). Addition of ML141 on aged MSCs enhances cell growth, plastic adherence, viability and decreases the senescence markers, p16, p21, p53 while it restores the balance between pro- and anti-inflammatory cytokines. Other treatments, including resveratrol and non-coding RNA modulation, may also reverse the altered phenotype in senescent MSCs (Okada et al., 2016; Vono et al., 2018).

## Impact of Circulating Senescent EVs on MSC Function

The SASP in local tissue microenvironment and in body fluids impacts the characteristics and functions of resident stem cells and participates to altered tissue homeostasis occurring with aging. EVs are proposed to take a large part in senescence induction as demonstrated in a number of studies, notably on MSCs. A first study reported that the miRNA profiles of EVs isolated from the bone marrow interstitial fluid (BMIF-EVs) from young or aged mice were different with a significant increase of the miR-183 cluster in aged samples (Davis et al., 2017). *In vitro*, aged BMIF-EVs were highly endocytosed by young MSCs, which displayed reduced capacity to differentiate into osteoblasts. Transfection of young MSCs with miR-183-5p reduced proliferation and osteogenesis while it increased senescence. Likewise, circulating EVs from blood plasma of elderly donors were shown to reduce the osteogenic potential of young MSCs (Weilner et al., 2016). MiR-31 produced by endothelial cells was detected at elevated levels in the plasma of osteoporotic and elderly patients and identified as one causal factor of osteogenesis inhibition by targeting Frizzled-3. Indeed, miR-31 may be involved in impaired bone formation in age-related diseases and may represent a valuable circulating biomarker for aging. Another study revealed that EVs from older women express high levels of C24:1 ceramide, a sphingolipid associated with the promotion of cell senescence and apoptosis, compared to younger individuals (Khayrullin et al., 2019). MSCs, which readily capture serum EVs, can be induced to senescence when EVs are loaded with C24:1 ceramide. In another report, muscle-derived circulating EVs isolated from the serum of old mice were shown to express higher levels of miR-34a, a miRNA associated with aging and inflammation, than in young mice (Fulzele et al., 2019). EVs recovered from miR-34a-overexpressing myoblasts reduced the survival of MSCs and increased senescence as detected by higher SA-βGal activity. Interestingly, EVs isolated from these myoblasts homed to bone *in vivo* and induced senescence of primary bone marrow-derived MSCs *ex vivo*. The authors concluded that aged skeletal muscle may be a potential source of circulating senescence-associated EVs impacting stem cell populations in tissues. Altogether, current data confirm that MSCs cultured *ex vivo* can be induced to senescence by circulating and tissue-derived aged EVs.

## Characteristics and Function of EVs From Senescent or Aged MSCs

Increase of EV production is a general feature in aged and senescence-induced cells as shown by several studies as early as 2008 (Lehmann et al., 2008; Beer et al., 2015; Takasugi et al., 2017). Accordingly, the production of EVs by MSCs increases with donor age and late passage cultures (Fafian-Labora et al., 2017, 2019; Lei et al., 2017). The release of EVs from senescent cells is at least partially dependent on p53 and its downstream target gene tumor suppression-activated pathway 6 (TSAP6). P53 is a transcriptional regulator of endosome-associated genes, including Rab5B and Rab27B, that play important roles in endosome regulation and exosome biosynthesis (Fujii et al., 2006). There are two possible explanations for the enhanced secretion of EVs from senescent cells. EVs mediate the removal of undesirable, misfolded and toxic molecules, notably cytoplasmic DNA, allowing survival of cells (for review, see Takahashi et al., 2017). Fragmented DNA is known to activate the DDR and the export of fragmented DNA by EVs may contribute to prevent the aberrant activation of DDR pathways. Alternatively, senescent cells release EVs in the surrounding environment as a protective mechanism to communicate a distress signal, enabling neighboring cells to react more rapidly and more efficiently to stress. However, it must be underlined that EVs may also represent a non-canonical part of the SASP contributing to a pro-senescent signal via a bystander effect.

**FIGURE 2:**
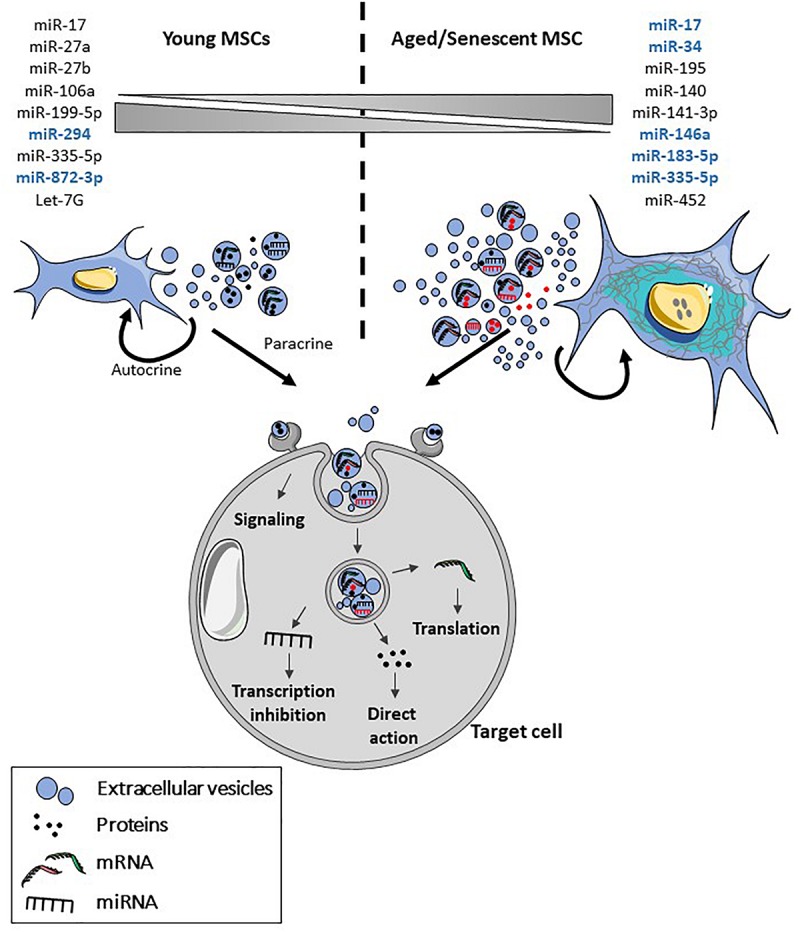
Features of senescent MSC-EVs. With aging and senescence, production of EVs by MSCs is increased and their cargo is altered. Both the content in different types of molecules and their number may be altered in MSCs. More specifically, several miRNAs have been identified as being modulated in MSCs and in MSC-EVs (blue) with aging and/or senescence. Upon release, MSC-EVs will interact in an autocrine and paracrine manner with the parental and target cells contributing to senescence propagation. Senescent MSC-EVs can interact directly with cell surface receptors and induce intracellular signaling pathways or fuse with the plasma membrane or be internalized by endocytosis. After internalization, proteins, miRNA and mRNA are released in the cytosol where they are functionally active.

A second feature of EVs released by senescent cells is altered cargos (for review, see Urbanelli et al., 2016). EVs are proposed to be involved in the modulation of chronic, systemic inflammation occurring during aging (inflamm-aging), which is associated with the progression of age-related diseases, through the transport of a number of miRNAs. These miRNAs called inflammamiRs regulate the main age-related processes: DDR, oxidative stress, proteotoxic stress, senescence or mitochondrial dysfunction (for a review, see Prattichizzo et al., 2017). A panel of inflammamiRs commonly identified in distinct cell types includes miR-19b, miR-20a, miR-21, miR-126, miR-146a, and miR-155. In MSC-EVs, expression of several miRNAs is modulated with increasing age (for review, see Fafian-Labora et al., 2017). A decreased expression of a number of miRNAs was observed in MSC-EVs from old versus young rats (Wang et al., 2015). A significant decrease was confirmed for miR-294 and miR-872-3p. In another study, miR-146a and miR-335-5p were up- and down-regulated, respectively, in late passage MSC-EVs versus early passage MSC-EVs but the expression of both miRNAs increased with increasing age in MSC-EVs (Lei et al., 2017). However, the modulated expression of these miRNAs in MSC-EVs was not demonstrated to be related to senescence induction. Finally, miR-183-5p was shown to be preferentially expressed in EVs isolated in bone marrow-derived MSCs from aged mice and to induce senescence features in young MSCs (Davis et al., 2017). The modulation of miRNAs in aged versus young MSC-EVs or late versus early passage MSC-EVs is illustrated in Figure 2. Furthermore, a number of miRNAs whose expression is modulated in aged versus young MSCs have been identified (Okada et al., 2016; Ganguly et al., 2017; Kulkarni et al., 2017; Vono et al., 2018; Figure 2). Their presence and expression level in MSC-EVs remain to be determined. However, two of them, miR-17 and miR-335-5p, have been described as higher in young MSCs versus aged MSCs and in aged MSC-EVs versus young MSC-EVs, respectively, suggesting a possible modulation of their expression in MSC-EVs with respect to the parental cells. These miRNAs identified in MSCs as senescence- and/or aging-associated factors have also been described in other cell types, except for miR-27b, miR-199-5p, miR-294, and miR-872-3p, highlighting their importance in the regulation of aging.

The current notion is that EVs exert similar functions as the parental cells. Indeed, the modulation of secretomes and EV contents released by senescent and/or aged MSCs likely contributes to their altered functions (for review, see Lunyak et al., 2017). Contrary to EVs isolated from aged MSCs, EVs from young MSCs were shown to rejuvenate old hematopoietic stem cells and restore their functions thanks to the transfer of autophagy- and lineage commitment-related mRNAs (Kulkarni et al., 2017). The authors discussed the hypothesis that reduced expression of these mRNAs in old MSC-EVs could be one of the mechanisms involved in niche-mediated aging of hematopoietic stem cells. Similarly, bone marrow MSC-EVs isolated from aged mice were reported to impair the sensitivity to insulin of adipocytes, myocytes and hepatocytes *in vitro* and to induce insulin resistance *in vivo*, through miR-29b-5p upregulation (Su et al., 2019). Another study described the therapeutic role of young MSC-EVs in liposaccharide-induced acute lung injury while aged MSC-EVs failed to exert protective effect (Huang et al., 2019). This effect was mediated by the switch in macrophage polarization toward an anti-inflammatory phenotype and an altered miRNA content. To our knowledge, no literature exists on the function of EVs isolated from senescence-induced MSCs. Interestingly, senescent fibroblasts isolated from oral submucous fibrosis biopsies were shown to participate in the improvement of fibrotic tissue through the secretion of MMPs (Pitiyage et al., 2011). Although we have to face to the lack of data on the contribution of EVs isolated from aged or senescent MSCs to the aging of the organism, the hypothesis that the secretome of senescent MSCs might influence and modulate stem cell niches and tissue homeostasis has been discussed elsewhere (for review, see Lunyak et al., 2017). Therefore, the possibility that senescent MSC-EVs may induce opposite effects depending on the tissue, the age or the context (inflammation, disease, …) has to be further investigated.

## Therapeutic Effect of MSC-EVs on Senescence in Age-Related Diseases

The regenerative properties of MSCs and MSC-EVs have been largely demonstrated and illustrated on a large variety of age-related degenerative diseases (for review, see Phinney and Pittenger, 2017; Chang et al., 2018; Chen et al., 2019). As examples, MSC-derived EVs were reported to have a chondroprotective effect in OA (Cosenza et al., 2017; D’Arrigo et al., 2019) and to exhibit beneficial role in type 1 and type 2 diabetes (Fan et al., 2019; Mahdipour et al., 2019), in cardiovascular diseases (Bian et al., 2014; Suzuki et al., 2017) or stroke (Doeppner et al., 2015).

The modulating role of EVs on the aging process has been demonstrated *in vivo* in a couple of studies. One of these studies reported that hypothalamic neural stem/progenitor cells (NSS) secrete decreasing amounts of EVs in the cerebrospinal fluid during aging (Zhang et al., 2017). Interestingly, a central treatment with healthy NSS-EVs could control whole body’s aging through the release of exosomal miRNAs. Another study showed that circulating levels of extracellular nicotinamide phosphoribosyltransferase (eNAMPT) decline with age and that over-expression of eNAMPT in adipose tissue or infusion of eNAMPT-containing EVs can extend the lifespan of aged mice (Yoshida et al., 2019). This effect was mediated by the release of eNAMPT-containing EVs into target cells that led to enhanced NAD^+^ synthesis, a known factor regulating the aging process. However, the effect of MSCs has been poorly investigated on age-associated senescence. One study reports that the secretome from human fetal MSCs ameliorates replicative senescence of adult MSCs as shown by significantly reduced SA-βGal expression and activity, enhanced cell proliferation and osteogenic differentiation potential in late passage (Wang et al., 2016). A similar approach demonstrated that the conditioned medium (CM) from MSCs regulated senescence features in IL1β-treated OA chondrocytes, namely SA-βGal activity, accumulation of γH2AX foci and reduction in the number of actin stress fibers (Platas et al., 2016). In addition, CM from MSCs decreased the oxidative stress, expression of p21 and enhanced the expression of sirtuin-1 (SIRT-1). These data were confirmed in another report showing that MSC-EVs from healthy donors downregulate SA-βGal activity and γH2AX foci in IL1β-treated osteoblasts isolated from OA patients (Tofino-Vian et al., 2017). MSC-EVs were also shown to reduce the production of the pro-inflammatory cytokines IL6 and PGE2 and the oxidative stress. Finally, EVs from young MSCs were reported to improve growth and to reduce senescent features of MSCs induced to genetic or replicative senescence (passages 10–14) (Liu et al., 2019).

## Conclusion and Perspectives

Aging impacts the function of MSCs and stimulates their senescence *in vivo*. With advancing donor age, senescent MSCs are characterized by a decline in the number of CFU-F, decreased capacity for differentiation, angiogenesis, wound healing properties and increased secretion of a SASP that contributes to senescence propagation. MSC-EVs have now been shown to be new components of the SASP and critical players in cellular senescence and aging. MSC-EVs are moving into the clinics for a number of therapeutic applications because this cell-free therapy offers more safety, better reproducibility and potentially higher scalability. Success of these therapies will depend on the physiological function of the parental cells and senescent MSCs may loss or have reduced therapeutic function and even worse, counteract the efficiency of the treatment. Aging therefore represents a limitation to the use of autologous MSCs, especially in older patients, for their use in tissue engineering and cell therapy applications. A better understanding of the senescence process will help controlling and modulating MSC-EVs cargos for boosting the beneficial effects of these innovative treatments.

## Author Contributions

All authors listed have made a substantial, direct and intellectual contribution to the work, and approved it for publication.

## Conflict of Interest

The authors declare that the research was conducted in the absence of any commercial or financial relationships that could be construed as a potential conflict of interest.
